# Psychometric Properties of the Oldenburg Burnout Inventory in a Portuguese Sample of Aircraft Maintenance Technicians

**DOI:** 10.3389/fpsyg.2021.725099

**Published:** 2021-12-16

**Authors:** Cátia Reis, Miguel Tecedeiro, Pollyana Pellegrino, Teresa Paiva, João P. Marôco

**Affiliations:** ^1^Universidade Católica Portuguesa, Católica Research Centre for Psychological - Family and Social Wellbeing, Lisbon, Portugal; ^2^Instituto de Medicina Molecular João Lobo Antunes, Faculdade de Medicina de Lisboa, Universidade de Lisboa, Lisboa, Portugal; ^3^Instituto de Saúde Ambiental, Faculdade de Medicina, Universidade de Lisboa, Lisboa, Portugal; ^4^William James Centre for Research, ISPA – Instituto Universitário, Lisboa, Portugal; ^5^UNISANTOS, Universidade Católica de Santos, Santos, Brasil; ^6^CENC – Sleep Medicine Center, Lisbon, Portugal; ^7^Faculdade de Ciências Médicas, Nova Medical School, CHRC, Lisbon, Portugal

**Keywords:** Oldenburg burnout inventory, aviation, validity, reliability, burnout, occupational stress

## Abstract

From its initial conceptualization as emotional exhaustion, cynicism, and reduced personal efficacy for the help professions, burnout has received increasing attention in modern times, especially after the 2019 WHO’s inclusion of this syndrome in the ICD-11 list. Burnout can be measured using several psychometric instruments that range in dimensionality, number of items, copyrighted, and free use formats. Here, we report the psychometric properties of data gathered with the Oldenburg Burnout Inventory (OLBI) in a sample of Portuguese Aircraft maintenance technicians. As far as we know, this is the first study addressing the burnout syndrome in this occupational group. Data gathered with the OLBI displayed good evidence of validity related to internal structure, to other variables, with good evidence of reliability. We showed that burnout significantly correlated with mental and physical fatigue emphasizing the vital critical role that these variables play with safety in the aviation industry.

## Introduction

Modern society has changed considerably over the last few years due to technology development and market globalization, which has imposed additional challenges to workers, usually reflected in higher stress levels (i.e., occupational, social, and physical stress). An important phenomenon associated with this development is the increase in shift work where “standard” day workers, that is, working a schedule between 07.30–8.00 and 17.00–18.00 h, from Monday to Friday, account only for approximately one-third of the global adult working force (Costa, 2003) with the implication that the majority of workers are working in “non-standard” schedules, like shifts. Shift work has been recognized as a physiological stressor since the workers are requested to perform in a time of the day where they were supposed to be sleeping (e.g., early morning, evening, or night shifts; Kecklund and Axelsson, 2016). Additionally, their social and family commitments need to be fulfilled and, consequently, these workers tend to develop sleep problems like sleep deprivation or insomnia, which in turn leads to increased levels of fatigue and lower performance (Åkerstedt, 2003; Vallières et al., 2014). Shift work is common in some sectors like health care, tourism, transports, and particularly the aviation field. Aircraft maintenance technician is an aviation job that involves the daily maintenance and repair of aircraft. Some repairs are programmed and must be performed in hangars, and others take place outdoors between flights (i.e., non-programmed; European Aviation Safety Agency, 2015). The latter ones are usually interventions involving troubleshooting under an enormous amount of stress, since the aircraft needs to be repaired as quickly as possible in order for the aircraft to continue to fly the planned schedule. This work is performed around the clock, often by workers working alone, and under adverse weather conditions (i.e., too hot, too cold, rain, and wind), with the need to access hard to reach places of the airplane to make the necessary repairs. The omnipresence of shift work in the aircraft maintenance sector generates, in the work force, levels of daytime sleepiness which are higher than the levels of sleep patients with obstructive sleep apnea, a medical sleep condition characterized by excessive levels of daytime sleepiness (Reis et al., 2020). The monitoring of this group of professionals is essential to prevent all possible hazards, such as aviation incidents or accidents. For airline pilots, work and rest times have been regulated (EASA, 2018), but there are no mandatory Rest & Recuperation norms for aircraft maintenance technicians, in spite of the fact that their job has a clear and direct impact on aircraft and flight safety. Furthermore, aeronautical technicians often work alone under extreme levels of time pressure, unlike airline pilots which, although having also high levels of stress, can share work and stress loads across the cockpit crew (captain and first officer). This makes these workers particularly vulnerable to occupational health problems (e.g., burnout).

The concept of burnout has been associated with human services professions (e.g., physicians, nurses, and teachers), but over the last years, this phenomenon has also been recognized for high physically and psychologically demanding jobs like professional athletes (Li et al., 2019) or airline pilots. A recent report on British airline pilots showed that 40% of participants presented high levels of burnout (Demerouti et al., 2019). As far as we know, no similar research has been conducted so far with aircraft maintenance technicians.

Burnout has become a global concern, and work-related stress is a big challenge to organizations. Burnout refers to a state of exhaustion and cynicism toward work and is one of the most popular research topics in occupational health psychology due to the recognized health problems and decreased performance associated with it. Burnout impacts not only the worker but also the organization, causing high levels of absenteeism, but also presenteeism (Demerouti et al., 2009), that is, the phenomena where employees report to work when they should be off sick. In some types of work (e.g., aviation and health care), this can create a safety issue jeopardizing not only the worker’s safety but also the safety of others.

Burnout was first introduced in the 1970s by psychologists (Freudenberger, 1974; Maslach, 1976) who conceptualized it as physical and emotional responses resulting from long-term job stress. In 1981, Maslach and Jackson conceptualized burnout as a three-dimension syndrome: a reduced sense of Personal Accomplishment, high emotional, physical and psychological Exhaustion, and high Depersonalization, a feeling of emotional distancing and indifference toward the recipients or beneficiaries of the work, affecting individuals who do “people-work” of any nature. Research showed that burnout could also be found in professional activities across all occupations, leading to Depersonalization being renamed Cynicism, an indifference and emotional distancing toward one’s work (Leiter and Schaufeli, 1996). Research also showed that emotional exhaustion and depersonalization/cynicism have a strong relationship with burnout levels, whereas personal accomplishment is poorly related to burnout levels and outcomes (Lee and Ashforth, 1996; Maslach et al., 2001; Demerouti et al., 2003; Shirom, 2003) leading burnout to be conceptualized as a two-dimension phenomena (Exhaustion and Disengagement/cynicism).

The oldest and best-known instrument to measure burnout is the Maslach Burnout Inventory (MBI; (Maslach et al., 1996). It follows the traditional three-factor structure of burnout, with burnout being expressed by a high score in emotional exhaustion, a high score in depersonalization, and a low score in personal accomplishment. The MBI has been criticized for issues relating to its construction, which in turn affects its psychometric properties (Lee and Ashforth, 1996; Demerouti et al., 2001; Bouman et al., 2002). The instrument is protected by copyright, requiring royalties to be paid for its use in research.

There are other valid options to measure burnout that can be used in any occupational group without any costs, options also better suited to the conceptualization of burnout as a two-factor construct. Examples of burnout measuring scales include the Bergen Burnout Inventory (BBI; Salmela-Aro et al., 2011); Copenhagen Burnout Inventory (CBI; Kristensen et al., 2005); Shirom-Melamed Burnout Questionnaire (SMBQ; Shirom and Melamed, 2006); the Karolinska Exhaustion Scale (KES; Saboonchi et al., 2013); and the Oldenburg Burnout Inventory (OLBI; Demerouti et al., 2003). OLBI was already translated and validated for several populations and to different languages like English (Halbesleben and Demerouti, 2005), Greek (Demerouti et al., 2003), Tamil (Subburaj and Vijayadurai, 2016), Malay (Mahadi et al., 2018), and Portuguese (Sinval et al., 2019). An adapted OLBI student version was already also validated for Greek and German (Reis et al., 2015), as well as for Portuguese (Campos et al., 2012). This instrument was already applied to different worker groups: health care (Peterson et al., 2008; Reis et al., 2015; Sprinks, 2015), students (Reis et al., 2015), teachers (Baka, 2015a; Innstrand, 2016), construction workers (Demerouti et al., 2010), executive directors (Olinske and Hellman, 2017), police officers (Baka, 2015b; Subburaj and Vijayadurai, 2016), bus drivers (Buitendach et al., 2016; Innstrand, 2016), and airline pilots (Demerouti et al., 2019). The diversity of working groups (ranging from blue to white collar workers) where OLBI was already used, presenting in general good values of internal consistency, makes OLBI a reliable instrument to use in different labor contexts.

In this paper, we studied the psychometric properties of the Portuguese version of the OLBI, namely, its validity evidence based on the internal structure (reliability, dimensionality, measurement invariance) and the validity evidence based on the relationship with other variables (depression, fatigue) in a sample of airline maintenance technicians. The search for sources of evidence supporting the psychometric properties of the OLBI in such a sample is a mandatory first step in a broader research goal of understanding burnout in aircraft maintenance crews and identifying factors impacted by burnout, such as poor health, depression, and memory lapses. We also hypothesize that burnout can predict worse health outcomes, namely, depression, as well as memory lapses for these workers.

### Research Hypotheses

Following the recommendations of the Standards for Educational and Psychological Testing (American Educational Research Association, 2014), this paper aims to assess two types of validity of the OLBI instrument in a sample of Portuguese aircraft maintenance technicians. This is a highly demanding profession in the aviation field where safety is a major issue. One validity evidence is related to the internal structure and the other based on the relationship to other variables. Various studies confirmed the original two-factor structure of OLBI in different occupation groups (Demerouti et al., 2010; Campos et al., 2012; Sinval et al., 2019). Therefore, we expected the Portuguese OLBI version to present a good fit, confirming its original dimensionality (two factors) for aircraft maintenance workers (H1). A second-order latent factor (burnout) was shown for the Portuguese version (Sinval et al., 2019), and this was also tested for this sample (H2). Other studies showed acceptable to very good reliability scores in terms of internal consistency, namely, for the Portuguese language study (CR = 0.93; a_ordinal_ = 0.93; ⍵ = 0.91), including subjects from different occupational groups from Portugal and Brazil (Sinval et al., 2019). Therefore, we predicted that both first-order OLBI dimensions (Exhaustion and Disengagement) and the second-order construct would present good internal consistency reliability estimates (H3).

Since exhaustion can be defined by intensive physical, affective, and cognitive strain, we hypothesized that physical and mental fatigue would be highly correlated to burnout, namely, to the exhaustion dimension of OLBI since it covers the physical and cognitive aspects of exhaustion (H4).

Another professional group from the aviation field performing a highly demanding job (airline pilots) presented high levels of burnout (Demerouti et al., 2019). Since burnout usually leads to a health detriment and is a moderator for daily function, we hypothesize that higher levels of depression and increased memory lapses could be predicted by burnout (H5) for the maintenance technicians’ occupational group. This might represent a risk factor for aviation safety since these workers can do line maintenance working alone, without the supervision and redundancy of a second technician, jeopardizing aircraft safety.

## Materials and Methods

### Participants

The total sample consisted of 348 Portuguese aviation maintenance technicians. These workers follow regular schedules or shift work schedules. These individuals answered a questionnaire composed of sociodemographic data, work data, and the following instruments: Oldenburg Burnout Inventory (OLBI; Sinval et al., 2019), Depression, Anxiety and Stress Scale (DASS-21; Pinto et al., 2015), and Multiple Dimension Fatigue Inventory (MFI; Smets et al., 1995).

Participation was anonymous, and all individuals gave their informed consent to participate in the study. The mean age of the total sample was 40.30 years (SD = 8.62) and ranged from 17 to 62 years. 96.3% were male. This percentage closely matches the male/female ratio among these workers. The educational level of the sample was: secondary level education: 67% and University education level: 33%. Of these workers, 14.7% work on a regular morning schedule and 85.3% were rotating shift workers. Regarding children, 70.1% had children, and individuals from both genders had small children living with them [χ^2^_(1)_ = 0.464; *p* = 0.491; *n* = 348]; 19.0% were single, 75.9% were married or with a partner, 4.6% were divorced, and 0.6% were widowed.

This was a convenience sample of Portuguese civil aircraft maintenance workers. The inclusion criteria were to be an aircraft technician for at least 6 months, from both sexes and from any educational level. The exclusion criteria was being on maternity/paternity leave, retired, or on sick leave.

### Measures

Burnout was assessed with the Portuguese version of the Oldenburg Burnout Inventory (OLBI; Sinval et al., 2019). This is a 16-item self-reported instrument with a five-point rating scale ranging from one = “Strongly disagree to five = “Strongly agree.” It understands burnout as a second-order factor that loads on two dimensions (disengagement and exhaustion), with eight items for each dimension (Demerouti et al., 2001, 2010). Disengagement refers to the distancing of workers from the work in general (content or object). Moreover, it represents the relationship between employees and their job, particularly their identification with the work that they are performing and the willingness to continue to perform that job. This dimension is represented by the following items: item 1 “*I always find new and interesting aspects in my work*”; item 3 “*It happens more and more often that I talk about my work in a negative way”; item 6 “Lately, I tend to think less at work and do my job in almost mechanically*”; item 7 “*I find my work to be a positive challenge*”; item 9 “*Over time, one can become disconnected from this type of work*”; item 11 “*Sometimes I feel sickened by my work tasks*”; *13* “*This is the only type of work I can imagine myself doing*” item 13 was removed from the Portuguese version due to the low loading, increasing internal consistency; and item 15 “*I feel more and more engaged in my work.*” Exhaustion is defined as a consequence of intensive physical, affective, and cognitive strain, that is, as a long-term consequence of prolonged exposure to certain job demands. This dimension is represented by the following items: item 2 “*There are days when I feel tired before I arrive at work*”; item 4 “*After work, I tend to need more time than in the past in order to relax and to feel better*”; item 5 “*I can tolerate the pressure of my work very well*”; item 8 “*During my work, I often feel emotionally drained*”; item 10 “*After working, I have enough energy for my leisure activity*”; item 12 “*After my work, I usually feel worn out and weary*”; item 14 “*Usually, I can manage the amount of my work well*”; and item 16 “*When I work, I usually feel energized.*”

Depression was assessed using the depression dimension of the Depression Anxiety Stress Scale (DASS-21; Pinto et al., 2015). This instrument measures three dimensions (depression, anxiety, and stress) using seven questions in each; thus, a total of 21 questions using a four-point rating scale to answer (0 – “*it does not apply to me at all*” to 3 – “*It applies to me most of the time*”). The Depression scale comprise questions evaluating the level of anhedonia (item 3 “*I could not seem to experience any positive feeling at all*”) and inertia (item 5 “*I found it difficult to work up the initiative to do things*”), discouragement (item 10 “*I felt that I had nothing to look forward to*”), dysphoria (item 13 “*I felt down-hearted and blue*”), lack of interest or involvement (item 16 “*I was unable to become enthusiastic about anything*”), self-depreciation (item 17 “*I felt I wasn’t worth much as a person*”), and devaluation of life (item 21 “*I felt that life was meaningless*”). In this study sample, the DASS-21 showed good psychometric properties for both Depression, Anxiety and Stress, as well as for the second-order General Distress factor (CFI = 0.976; NFI = 0.955; TLI = 0.976; SRMR = 0.067; RMSEA = 0.055, a = 0.747 to 0.880). In this study, we only used the depression dimension (α = 0.914; and ω = 0.875).

Individual levels of mental and physical fatigue were measured using the Portuguese version of the Multiple Dimension Fatigue Inventory. This self-report instrument has five dimensions (general fatigue, physical fatigue, mental fatigue, reduced activity, and reduced motivation), each containing four items. Among these four items, two are for the measured factor and two are against (e.g., “*I feel fit*” and “*Physically I feel only able to do a little*”), thus trying to obtain the closest answer to reality, reducing response tendencies. In the total of 20 items, the participants are asked to tick one of the five response boxes ranging from agreement (“*yes, that is true*”) to disagreement with the statement (“*no, that is not true*”). This instrument was chosen because it has a clear distinction between mental (e.g., item seven “*When I am doing something, I can keep my thoughts on it*”) and physical fatigue (e.g., item two “*Physically I feel only able to do a little*”), and we used only the dimensions of mental fatigue and physical fatigue to assess concurrent validity (Barrett et al., 1981). Burnout is considered as a state of psychological exhaustion, as so, it is expected that burnout associates positively with fatigue, namely with mental fatigue. This instrument showed good psychometric properties (CFI = 0.973; NFI = 0.966; TLI = 0.955; SRMR = 0.053; RMSEA = 0.102; physical fatigue α = 0.774; ω = 0.771 and mental fatigue α = 0.825; ω = 0.741).

Since impaired daily functioning was shown to be associated with burnout (Bakker and Costa, 2014), as a proxy for impaired daytime functioning and mental performance we asked whether the participants experienced memory lapses “*Do you use to have small lapses of memory like forgetting or losing the car keys?*.” This variable was categorized as *yes* or *no*.

### Procedures

Data were collected between May and June 2019 recurring to a web survey. The study objectives were clearly explained before starting the survey, and it was explained that they could stop answering whenever they want and that anonymity was ensured since no identification data was requested, and a code was attributed to each participant. The informed consent was a mandatory question in order to start the questionnaire. Since web surveys on average present low response rates (Massey and Tourangeau, 2013), advertisements *via* email and by mobile phone text message were sent by the SITEMA (worker’s association) asking for their participation. For a total of 600 workers, we had 348 valid responses, obtaining a response rate of 60%. The estimated time for response was established in approximately 30–40 min.

The study was approved by the Ethics Committee of the Lisbon Medical School, Portugal (Reference number 190/19).

### Data Analysis

Items’ psychometric properties were assessed using descriptive statistics [mean, mode, Standard Deviation (SD), skewness (sk), and kurtosis (ku)] calculated by the *skimr* library (McNamara et al., 2018) for the R statistical system (R Core Team, 2018). It was assumed that items with absolute values of sk and ku larger than two and seven were indicative of severe violations to the normal distribution assumption that would recommend against its use in structural equation modeling (SEM) with regular maximum likelihood estimation (Finney and DiStefano, 2013; Marôco, 2021). Evidence related to the internal structure (construct-related validity) was studied by means of confirmatory factor analysis (CFA) on the polychoric correlation matrix using the diagonally weighted least squares (DWLS) estimator implemented in the *lavaan* package (Rosseel, 2012). Items with standardized factor loadings smaller than 0.04 were discarded (Fornell and Larcker, 1981; Marôco, 2021). Comparative fit index (CFI), Tucker-Lewis index (TLI), standardized root mean square residual (SRMR), and root mean square error of approximation (RMSEA) were used as goodness-of-fit indices. CFI and TLI above 0.9, as well as SRMR and RMSEA below 0.08 were indicative of good model fit (Byrne, 2010; Marôco, 2021). The *lavaan* package was also used to probe sources of evidence related to other variables by means of correlational analysis of the OLBI with mental and physical fatigue as well as regression on depression and memory lapses. For the memory lapses regression on Burnout, a Probit model was used since memory lapses is a dichotomous variable (yes/no). Average variance extracted (AVE), Cronbach’s ordinal α, and McDonald’s ordinal ω were used to assess reliability. Discriminant validity was assessed by both Fornell and Larcker (1981) criterion of AVE for two factors larger than the squared correlation between the factors and by the heterotrait-monotrait ratio of correlations (HTMT). This later method has been more recently proposed as a better alternative criterion to probe discriminant validity when using SEM results (Henseler et al., 2015). AVE and HTMT were estimated using the semTools package (Jorgensen et al., 2021). AVE above 0.5 was indicative of good evidence of convergent validity (Fornell and Larcker, 1981; Marôco, 2021), while a and ω above 0.7 were indicative of good reliability (Marôco, 2021), and HTMT values below 0.9 are indicative of discriminant validity evidence (Henseler et al., 2015). Finally, invariance for the OLBI measurement model was pursued by comparing a series of nested models ranging from no restrictions to the measurement model between groups (configural invariance), equal factor loadings (metric or week invariance), equal intercepts/thresholds (strong or scalar invariance), equal factor means (strong means invariance); and equal residuals variance (strict invariance). Invariance analysis for education (Secondary vs. University) and shift workers (yes/no) was performed using the package equaltestMI (Jiang and Mai, 2021) with robust maximum likelihood estimation. Invariance between nested models was assumed for non-significand Δχ^2^ between two consecutive nested models or absolute ΔCFI smaller than 0.01 (Cheung and Rensvold, 2002) and ΔRMSEA smaller than 0.02 (Rutkowski and Svetina, 2014).

## Results

### Items’ Distributional Properties

The items’ distribution properties are shown in Table 1. Summary measures, skewness, kurtosis, and a histogram of the 16 items is presented. These items follow an approximately normal distribution for the studied population since their distributional properties, judging from the sk and ku values, are suggestive of appropriate psychometric sensitivity.

**Table 1:** Item descriptive statistics (*n* = 348).

OLBI items	Mean	SD	P1	P25	P50	P75	P99	Sk	Ku	Histogram
OLBI 1^D^	2.58	0.92	1	2	2	3	5	0.64	0.06	▁▇▅▂▁
OLBI 2^E^	3.82	0.83	1	4	4	4	5	−1.00	1.45	▁▁▂▇▂
OLBI 3^D^	3.03	1.08	1	2	3	4	5	−0.04	−0.87	▂▇▇▇▂
OLBI 4^E^	3.70	0.95	1	3	4	4	5	−0.74	0.29	▁▂▃▇▃
OLBI 5^E^	2.22	0.68	1	2	2	3	5	0.69	1.42	▁▇▃▁▁
OLBI 6^D^	2.86	1.03	1	2	3	4	5	0.02	−0.78	▂▇▇▇▁
OLBI 7^D^	2.26	0.88	1	2	2	3	5	0.84	0.78	▂▇▃▁▁
OLBI 8^E^	3.16	1.07	1	2	3	4	5	−0.12	−0.80	▂▆▇▇▂
OLBI 9^D^	2.77	1.09	1	2	3	4	5	0.12	−0.82	▃▇▇▆▁
OLBI 10^E^	3.00	0.94	1	2	3	4	5	0.15	−0.59	▁▆▇▆▁
OLBI 11^D^	3.14	1.06	1	2	3	4	5	−0.24	−0.78	▂▅▆▇▂
OLBI 12^E^	3.33	0.95	1	3	3	4	5	−0.16	−0.63	▁▅▇▇▂
OLBI 13^D^	3.04	1.21	1	2	3	4	5	0.02	−0.90	▃▆▇▆▃
OLBI 14^E^	2.32	0.77	1	2	2	3	5	0.80	1.06	▁▇▃▁▁
OLBI 15^D^	3.05	0.89	1	2	3	4	5	0.21	−0.14	▁▃▇▃▁
OLBI 16^E^	2.79	0.84	1	2	3	3	5	0.49	0.03	▁▇▇▃▁

D, disengagement items; E, exhaustion items.

### Validity Evidence Based on Internal Structure

**Figure 1:**
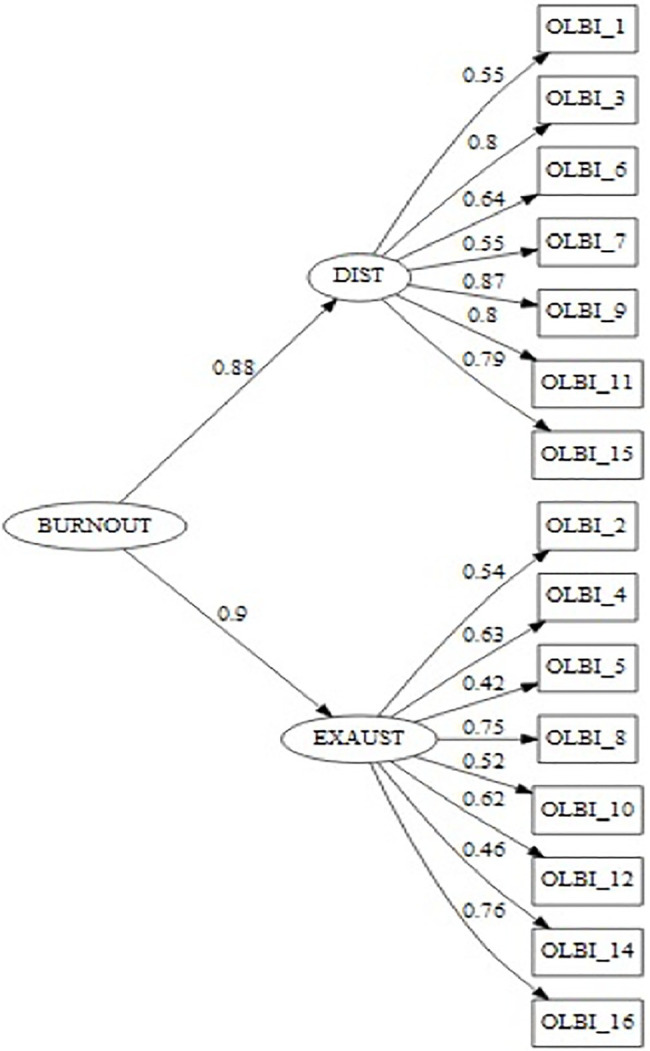
OLBI’s second-order model factor reduced version (15 items) structure fit for the sample of aeronautic mechanic technicians (*n* = 348). Numeric values are the factor loadings for each first-order factors and their respective items [χ^2^_(86)_ = 363.461; *p* < 0.001; n = 348; CFI = 0.927; NFI = 0.955; TLI = 0.983; SRMR = 0.064; RMSEA = 0.096; P(RMSEA ≤ 0.05) < 0.001; 90% CI(0.086; 0.107)].

Confirmatory factor analysis was performed for the 16 items. Item 13 had a very low factorial weight (𝜆_item 13_ = 0.222), and thus, just like for a previous validated Portuguese version (Sinval et al., 2019), this item was removed from further analysis. Factor loadings for the first-order model are shown in Table 2. We confirm the good goodness-of-fit indices for the reduced 15-item version. The OLBI’s second-order latent factor model presented an acceptable fit [χ^2^_(86)_ = 363.461; *p* < 0.001; *n* = 348; CFI = 0.927; NFI = 0.955; TLI = 0.983; SRMR = 0.064; RMSEA = 0.096; P(RMSEA ≤ 0.05) < 0.001; 90% CI (0.086; 0.107)]. For the respective loading factors, see Table 2. The two-factor structure of the OLBI (see Figure 1) was also confirmed for the Portuguese sample of aeronautical technicians as well as the second-order latent factor (burnout).

**Table 2:** Factor loadings for first-order model.

Latent factor	Indicator	Beta	SE	Z	sig
Disengagement	OLBI 1	0.55	0.04	13.53	*p* < 0.001
	OLBI 3	0.80	0.02	32.59	*p* < 0.001
	OLBI 6	0.64	0.03	19.11	*p* < 0.001
	OLBI 7	0.55	0.04	13.00	*p* < 0.001
	OLBI 9	0.87	0.02	46.24	*p* < 0.001
	OLBI 11	0.80	0.02	34.81	*p* < 0.001
	OLBI 15	0.79	0.03	31.66	*p* < 0.001
Exhaustion	OLBI 2	0.54	0.04	14.31	*p* < 0.001
	OLBI 4	0.63	0.03	18.48	*p* < 0.001
	OLBI 5	0.42	0.06	7.65	*p* < 0.001
	OLBI 8	0.75	0.03	24.25	*p* < 0.001
	OLBI 10	0.52	0.04	12.68	*p* < 0.001
	OLBI 12	0.62	0.04	17.34	*p* < 0.001
	OLBI 14	0.46	0.05	9.57	*p* < 0.001
	OLBI 16	0.76	0.03	25.15	*p* < 0.001

### Convergent and Discriminant Validity

To account for convergent validity, and in order to check whether each factor relates to their respective items, the average variance extracted (AVE) was calculated for disengagement (AVE = 0.384) and for exhaustion (AVE = 0.474). These results suggest no convergent validity for the reduced 15 item OLBI.

The heterotrait-monotrait (HTMT) ratio of correlations method (Henseler et al., 2015) was used to assess discriminant validity, and the value obtained was of 0.723. This result suggests a good discriminant validity. However, using the Fornell and Larcker (1981) criterion, the AVE were 0.474 for Disengagement and 0.384 for Exhaustion which are both smaller than the squared correlation between these two factors (*r*^2^ = 0.565) suggesting poor discriminant validity of the two first-order factors. HTMT has been shown to be upward biased if the measurement of the latent variables is not τ-equivalent and the correlation between the latent variables approaches one (Roemer et al., 2021).

### Reliability

The reliability estimates for the first-order factors were as follows: disengagement (Cronbach’s ordinal α = 0.883; and McDonalds’ ω = 0.853) and exhaustion (Cronbach’s ordinal α = 0.816; and McDonalds’ ω = 0.756). The reliability estimate for 2nd-order burnout factor was ω = 0.785.

### Validity Evidence Based on the Relations to Other Variables

Burnout can be considered as an increased feeling of emotional exhaustion (Maslach and Jackson, 1981) as so, in order to assess concurrent validity, we performed the correlation between the second-order model of the instrument (burnout), and the first-order latent variables (disengagement and exhaustion) and the dimensions of physical and mental fatigue of the MFI instrument (Table 3). The obtained correlation between variables were positive and ranging from medium to strong, demonstrating a good concurrent validity.

**Table 3:** Correlations between OLBI and physical and mental fatigue.

	Mental fatigue	Physical fatigue
Mental fatigue	1	-
Physical fatigue	0.477	1
Disengagement	0.457	0.407
Exhaustion	0.554	0.595
Burnout	0.570	0.565

*All correlations were statistically significant for p < 0.001*.

An overlap between depression and burnout has been described since the development of the burnout construct: Freudenberger in 1974 reported that a person suffering from burnout seems depressed (Freudenberger, 1974). Based on this, regression analysis showed the predictive value of burnout for depression (β = 0.733, *p* < 0.001, R^2^ = 0.533 *p* < 0.001).

Considering the impaired daytime function associated with burnout, the predictive value of burnout for memory lapses was also tested. A strong predictive validity of burnout on memory lapses was observed (Probit β = 0.487, *p* < 0.001, R^2^ = 0.237 *p* < 0.001). These results both confirmed H5.

### Measurement Invariance for Education Level and Shiftwork

Measurement invariance was probed for educational level (Secondary education vs. University degree). There were metric [Δχ^2^(13) = 14.443; *p* = 0.307, ΔCFI = 0.001, ΔRMSEA = 0.003], scalar[Δχ^2^(12) = 14.443; *p* = 0.273, ΔCFI = 0.001, ΔRMSEA = 0.003], strong means [Δχ^2^(3) = 5.996; *p* = 0.112, ΔCFI = 0.001, ΔRMSEA = 0.000], and strict [Δχ^2^(15) = 11.955; *p* = 0.682, ΔCFI = 0.002, ΔRMSEA = 0.003] invariance for workers with secondary education and workers with a university education. Similarly, metric [Δχ^2^(13) = 12.506; *p* = 0.487, ΔCFI = 0.000, ΔRMSEA = 0.003], scalar [Δχ^2^(12) = 14.5950; *p* = 0.166, ΔCFI = 0.002, ΔRMSEA = 0.002], strong means [Δχ^2^(3) = 4.312; *p* = 0.229, ΔCFI = 0.001, ΔRMSEA = 0.000], and strict [Δχ^2^(15) = 5.9631; *p* = 0.980, ΔCFI = 0.005, ΔRMSEA = 0.005] invariance was observed between workers who worked in shifts and workers who did not work in shifts.

## Discussion

The present study examines the OLBI’s psychometric properties for a specific group of workers of the aviation field. The 2-factor structure (exhaustion and disengagement) of the OLBI was shown to have an adequate fit in this sample, suggesting that this structure is essentially invariant across Portuguese-speaking occupational groups, like previously reported (Sinval et al., 2019). The obtained results are in line with the original OLBI validation study performed by Demerouti and collaborators for a sample of Greek workers from different sectors (CFI = 0.95, GFI = 0.94, RMSEA = 0.062). The scale showed poor discriminant validity between the two first-order factors (Exhaustion and Disengagement) which provided empirical support for the existence of the second-order Burnout factor. Item 13 was removed from this version due to the low factor loading, increasing the internal consistency of the instrument, like previously reported for Portuguese and Brazilian general versions, where good evidence of validity was observed (e.g., CFI = 0.986, TLI = 0.983, SRMR = 0.064, RMSEA = 0.093; Sinval et al., 2019) as well as for the students’ version (CFI = 0.913, GFI = 0.944, RMSEA = 0.058; Campos et al., 2012). This item has been also removed from the Italian (Estévez-Mujica and Quintane, 2018) and Russian (CFI = 0.95, RMSEA = 0.03; Smirnova, 2017) versions for the same reason. The consistency of these results might suggest that item 13 could be dropped from the scale. The results from these studies confirm the overall good validity evidence of the OLBI across different occupational groups. Our results add to this validity evidence for aircraft maintenance technicians.

OLBI presented a good internal consistency for aeronautical technicians with a good reliability of the second-order latent factor model like previously reported for other groups of Portuguese-speaking workers as well as for other language versions (Cronbach’s α ranging from 0.69 to 0.91 for Disengagement, and from 0.85 to 0.87 for Exhaustion; Smirnova, 2017; Estévez-Mujica and Quintane, 2018; Sinval et al., 2019).

OLBI displayed strong invariance for both education level and shiftwork. Thus, the OLBI can be used to produce valid and reliable data for aircraft maintenance technicians with either secondary education or university education. Reading and comprehension of the OLBI items does not require a university education. Similarly, the OLBI can be used to assess Burnout in shift workers and non-shift workers.

### Practical Implications

OLBI showed to be a reliable measure to access burnout for this specific group of workers. Aircraft maintenance work is rated as an area of high responsibility for its potential dangers since there is a close link between standards of maintenance and aircraft safety. In this study, burnout could predict memory losses and this lower cognitive performance while performing aircraft maintenance can represent a threat to aviation safety. As such, it is highly important to monitor and prevent burnout symptoms for these workers. The Portuguese OLBI version showed to be reliable, free of cost, and an easy to apply instrument to measure burnout in this professional group. Another practical implication of assessing burnout preventively for these workers was also shown in this report since burnout could also predict depressive symptoms. These were already associated with a decreased performance at work, increasing the odds of on duty accidents to happen (Haslam, 2005; Tsuchiya et al., 2012).

In this report, we could observe that safety issues might be present for these workers due to the existence of burnout symptoms. The implementation of redundancy protocols and specific working rules, like the ones established for airline pilots, could be an important measure, such as adding the mandatory presence of two maintenance elements, namely, for repair work done between flights, since these are the ones presenting higher stress levels, increasing the propensity for errors and jeopardizing aviation safety.

### Limitations

This study used a convenience sample only composed by Portuguese aircraft technicians. This study had very few women, basically representing the ratio for men and women for this professional group, and this factor did not allow a proper sex comparison validation for these workers. In any case, we could see that burnout scores were equally distributed in both genders [χ ^2^_(1)_ = 0.020; *p* = 1.000; *N* = 348] and the instrument invariance for sex was already reported for other professional groups and namely for a sample of Portuguese-speaking workers (Sinval et al., 2019). There were other studies where these differences were present although they were associated with other factors like family and workload (Bekker et al., 2005; Langballe et al., 2011). We also did not probe for temporal stability of the OLBI or its evidence of concurrent validity with other Burnout measures. However, evidence of concurrent validity of the OLBI with the MBI-GS has been demonstrated by Halbesleben and Demerouti (2005) for a general American working population and by Campos et al. (2012) for Portuguese and Brazilian students.

### Future Research

Since this study was composed only by Portuguese aircraft maintenance workers, it would be important to test the invariance between countries for other Portuguese-speaking populations (e.g., Brazil and Angola). To test OLBI’s psychometric properties for other groups from the aviation field, like airline pilots, would be also highly relevant since they are also critical workers of the aviation industry, presenting reported high levels of fatigue and sleep problems (Reis et al., 2013, 2016), although with higher control of work and rest periods, as well as redundancy protocols (EASA, 2018).

## Conclusion

The reduced OLBI version showed to be a good instrument to assess burnout for a sample of aircraft maintenance technicians, a highly demanding technical profession that performs under enormous amounts of stress and where the surveillance of burnout levels is important in order to avoid safety issues.

## Data Availability Statement

The original contributions presented in the study are included in the article/supplementary material, and further inquiries can be directed to the corresponding author.

## Ethics Statement

The studies involving human participants were reviewed and approved by Ethics Committee of the Lisbon Medical School, Portugal (Reference number 190/19). The patients/participants provided their written informed consent to participate in this study.

## Author Contributions

CR, PP, TP, and JM: conceptualization and methodology. CR and PP: data collection. CR, MT, and JM: formal analysis and writing original draft preparation. JM: supervision. All authors reviewed and approved the final version of the manuscript.

## Funding

PhD fellowship - PP - Coordenação de Aperfeiçoamento de Pessoal de Nível Superior, Grant/Award, Number: 88887150178/2017-00.

## Conflict of Interest

The authors declare that the research was conducted in the absence of any commercial or financial relationships that could be construed as a potential conflict of interest.

## Publisher’s Note

All claims expressed in this article are solely those of the authors and do not necessarily represent those of their affiliated organizations, or those of the publisher, the editors and the reviewers. Any product that may be evaluated in this article, or claim that may be made by its manufacturer, is not guaranteed or endorsed by the publisher.

## References

[ref1] Åkerstedt T. (2003). Shift work and disturbed sleep/wakefulness. Occupational Medicine 2, 117–28. Available at: http://www.ncbi.nlm.nih.gov/pubmed/15310506 (Accessed August 4, 2013).

[ref2] American Educational Research Association (2014). Standards for educational and psychological testing. eds. American Educational Research Association, American Psychological Association, and National Council on Measurement in Education.

[ref3] Baka Ł. (2015a). Does job burnout mediate negative effects of job demands on mental and physical health in a group of teachers? Testing the energetic process of job demands-resources model. Int. J. Occup. Med. Environ. Health 28, 335–346. doi: 10.13075/ijomeh.1896.00246, PMID: 26182928

[ref4] Baka L. (2015b). The effects of job demands on mental and physical health in the groups of police officers. Testing the ediating role of job burnout. Stud. Psychol. 57, 285–300. doi: 10.21909/sp.2015.03.700

[ref5] Bakker A. B. Costa P. L. (2014). Chronic job burnout and daily functioning: A theoretical analysis. Burn. Res. 1, 112–119. doi: 10.1016/j.burn.2014.04.003

[ref601] Barrett G. V. Phillips J. S. Alexander R. A. (1981). Concurrent and predictive validity designs: A critical reanalysis. J. Appl. Psychol. 66, 1–6.

[ref6] Bekker M. H. J. Croon M. A. Bressers B. (2005). Childcare involvement, job characteristics, gender and work attitudes as predictors of emotional exhaustion and sickness absence. Work Stress. 19, 221–237. doi: 10.1080/02678370500286095

[ref602] Bouman A. M. Te Brake H. Hoogstraten J. (2002). Significant effects due to rephrasing the Maslach Burnout Inventory’s personal accomplishment items. Psychol. Rep. 91, 825–826. doi: 10.2466/PR0.91.7.825-826 12530729

[ref7] Buitendach J. H. Bobat S. Muzvidziwa R. F. Kanengoni H. (2016). Work engagement and its relationship with various dimensions of work-related well-being in the public transport industry. Psychol. Dev. Soc. 28, 50–72. doi: 10.1177/0971333615622895

[ref8] Byrne B. M. (2010). Structural Equation Modeling with AMOS. 3rd *ed.* New York, NY: Routledge.

[ref9] Campos J. A. D. B. Carlotto M. S. Marôco J. (2012). Oldenburg burnout inventory - student version: cultural adaptation and validation into Portuguese. Psicol.: Reflex. Crit 25, 709–718. doi: 10.1590/S0102-79722012000400010

[ref10] Cheung G. W. Rensvold R. B. (2002). Evaluating goodness-of-fit indexes for testing measurement invariance. Struct. Equ. Model. Multidiscip. J. 9, 233–255. doi: 10.1207/S15328007SEM0902_5, PMID: 22551991

[ref11] Costa G. (2003). Shift work and occupational medicine: an overview. Occup. Med. 53, 83–88. doi: 10.1093/occmed/kqg045, PMID: 12637591

[ref12] Demerouti E. Bakker A. B. Nachreiner F. Schaufeli W. B. (2001). The job demands-resources model of burnout. J. Appl. Psychol. 86, 499–512. doi: 10.1037/0021-9010.86.3.499, PMID: 11419809

[ref13] Demerouti E. Bakker A. B. Vardakou I. Kantas A. (2003). The convergent validity of two burnout instruments: a multitrait-multimethod analysis. Eur. J. Psychol. Assess. 19, 12–23. doi: 10.1027//1015-5759.19.1.12

[ref14] Demerouti E. le Blanc P. M. Bakker A. B. Schaufeli W. B. Hox J. (2009). Present but sick: a three-wave study on job demands, presenteeism and burnout. Career Dev. Int. 14, 50–68. doi: 10.1108/13620430910933574

[ref15] Demerouti E. Mostert K. Bakker A. B. (2010). Burnout and work engagement: A thorough investigation of the independency of both constructs. J. Occup. Health Psychol. 15, 209–222. doi: 10.1037/a0019408, PMID: 20604629

[ref16] Demerouti E. Veldhuis W. Coombes C. Hunter R. (2019). Burnout among pilots: psychosocial factors related to happiness and performance at simulator training. Ergonomics 62, 233–245. doi: 10.1080/00140139.2018.1464667 29648499

[ref17] EASA (2018). Effectiveness of Flight Time Limitation (FTL). 11. Available at: https://www.easa.europa.eu/document-library/general-publications/effectiveness-flight-time-limitation-ftl-report#group-easa-downloads (Assessed June 12, 2021).

[ref18] Estévez-Mujica C. P. Quintane E. (2018). E-mail communication patterns and job burnout. PLoS One 13:e0193966. doi: 10.1371/journal.pone.0193966, PMID: 29518128 PMC5843271

[ref19] European Aviation Safety Agency (2015). Acceptable Means of Compliance (AMC) and Guidance Material (GM) Annex III (PART-66) to Regulation (EU) No 1321/2014. Available at: https://www.easa.europa.eu/document-library/regulations/commission-regulation-eu-no-13212014 (Accessed June 14, 2021).

[ref20] Finney S. J. DiStefano C. (2013). in Non-Normal and Categorical Data in Structural Equation Modeling. eds. Hancock Mueller R. O.. 2nd *ed* (Charlotte, NC: Information Age Publishing).

[ref21] Fornell C. Larcker D. F. (1981). Evaluating structural equation models with unobservable variables and measurement error. J. Mark. Res. 18, 39–50. doi: 10.1177/002224378101800104

[ref22] Freudenberger H. J. (1974). Staff Burn-Out. J. Soc. Issues 30, 159–165. doi: 10.1111/j.1540-4560.1974.tb00706.x, PMID: 34744464

[ref23] Halbesleben J. R. B. Demerouti E. (2005). The construct validity of an alternative measure of burnout: investigating the English translation of the Oldenburg burnout inventory. Work Stress. 19, 208–220. doi: 10.1080/02678370500340728

[ref24] Haslam C. (2005). Perceptions of the impact of depression and anxiety and the medication for these conditions on safety in the workplace. Occup. Environ. Med. 62, 538–545. doi: 10.1136/oem.2004.016196, PMID: 16046606 PMC1741070

[ref25] Henseler J. Ringle C. M. Sarstedt M. (2015). A new criterion for assessing discriminant validity in variance-based structural equation modeling. J. Acad. Mark. Sci. 43, 115–135. doi: 10.1007/s11747-014-0403-8

[ref26] Innstrand S. T. (2016). Occupational differences in work engagement: A longitudinal study among eight occupational groups in Norway. Scand. J. Psychol. 57, 338–349. doi: 10.1111/sjop.12298, PMID: 27263496

[ref27] Jiang Ge Mai Yujiao (2021). equaltestMI: Examine Measurement Invariance via Equivalence Testing and Projection Method. R package version 0.6.1. Available at: https://CRAN.R-project.org/package=equaltestMI (Accessed September 6, 2021).

[ref28] Jorgensen T. D. Pornprasertmanit S. Schoemann A. M. Rosseel Y. (2021). semTools: Useful tools for structural equation modeling. R package version 0.5–4. Available at: https://CRAN.R-project.org/package=semTools (Accessed June 11, 2021).

[ref29] Kecklund G. Axelsson J. (2016). Health consequences of shift work and insufficient sleep. BMJ 355:i5210. doi: 10.1136/bmj.i5210, PMID: 27803010

[ref30] Kristensen T. S. Borritz M. Villadsen E. Christensen K. B. (2005). The Copenhagen burnout inventory: A new tool for the assessment of burnout. Work Stress. 19, 192–207. doi: 10.1080/02678370500297720, PMID: 32067408

[ref31] Langballe E. M. Innstrand S. T. Aasland O. G. Falkum E. (2011). The predictive value of individual factors, work-related factors, and work-home interaction on burnout in female and male physicians: a longitudinal study. Stress. Health 27, 73–85. doi: 10.1002/smi.1321

[ref32] Lee R. T. Ashforth B. E. (1996). A meta-analytic examination of the correlates of the three dimensions of job burnout. J. Appl. Psychol. 81, 123–133. doi: 10.1037/0021-9010.81.2.123, PMID: 8603909

[ref33] Leiter M. P. Schaufeli W. B. (1996). Consistency of the burnout construct across occupations. Anxiety Stress Coping 9, 229–243. doi: 10.1080/10615809608249404, PMID: 9476708

[ref34] Li C. Zhu Y. Zhang M. Gustafsson H. Chen T. (2019). Mindfulness and athlete burnout: A systematic review and meta-analysis. Int. J. Environ. Res. Public Health 16:449. doi: 10.3390/ijerph16030449, PMID: 30717450 PMC6388258

[ref35] Mahadi N. F. Chin R. W. A. Chua Y. Y. Chu M. N. Wong M. S. Yusoff M. S. B.. (2018). Malay language translation and validation of the Oldenburg burnout inventory measuring burnout. Educ. Med. J. 10, 27–40. doi: 10.21315/eimj2018.10.2.4

[ref36] Marôco J. (2021). Análise de Equações Estruturais, Fundamentos teóricos, Software e aplicações. 3 edição. Pêro Pinheiro: Report Numbers.

[ref37] Maslach C. (1976). Burned-out. Hum. Behav. 9, 16–22.

[ref38] Maslach C. Jackson S. E. (1981). The measurement of experienced burnout. J. Organ. Behav. 2, 99–113. doi: 10.1002/job.4030020205, PMID: 33588654

[ref39] Maslach C. Jackson S. Leiter M. (1996). Maslach Burnout Inventory Manual (3rd *ed.*). Mountain View, CA: CPP, Inc.

[ref40] Maslach C. Schaufeli W. B. Leiter M. P. (2001). Job burnout. Annu. Rev. Psychol. 52, 397–422. doi: 10.1146/annurev.psych.52.1.397, PMID: 11148311

[ref41] Massey D. S. Tourangeau R. (2013). Introduction: new challenges to the social measurement. Ann. Am. Acad. Pol. Soc. Sci. 645, 6–22. doi: 10.1177/0002716212463314, PMID: 25506081 PMC4263208

[ref42] McNamara A. Arino de la Rubia E. Zhu H. Ellis S. Quinn M. (2018). skimr: Compact and Flexible Summaries of Data (R package version 1.0.3) [Computer software]. Available at: https://cran.r-project.org/package=skimr (Accessed June 11, 2021).

[ref43] Olinske J. L. Hellman C. M. (2017). Leadership in the human service nonprofit organization: The influence of the Board of Directors on executive director well-being and burnout. Hum. Serv. Organ. Manag. Leadersh. Gov. 41, 95–105. doi: 10.1080/23303131.2016.1222976

[ref44] Peterson U. Bergström G. Samuelsson M. Åsberg M. Nygren Å. (2008). Reflecting peer-support groups in the prevention of stress and burnout: randomized controlled trial. J. Adv. Nurs. 63, 506–516. doi: 10.1111/j.1365-2648.2008.04743.x, PMID: 18727753

[ref45] Pinto J. Martins P. Pinheiro T. Oliveira A. (2015). Anxiety, depression and stress: a study of portuguese adults. Psicologia, Saúde & Doenças 16, 148–163. doi: 10.15309/15psd160202

[ref46] R Core Team (2018). R: A Language and Environment for Statistical Computing (version 4.0) [Computer software]. Available at: https://www.r-project.org/ (Accessed June 11, 2021).

[ref47] Reis C. Mestre C. Canhão H. (2013). Prevalence of fatigue in a group of airline pilots. Aviat. Space Environ. Med. 84, 828–833. doi: 10.3357/ASEM.3548.2013, PMID: 23926658

[ref48] Reis C. Mestre C. Canhão H. Gradwell D. Paiva T. (2016). Sleep complaints and fatigue of airline pilots. Sleep Sci 9, 73–77. doi: 10.1016/j.slsci.2016.05.003, PMID: 27656269 PMC5021958

[ref49] Reis C. Staats R. Pellegrino P. Alvarenga T. A. Bárbara C. Paiva T. (2020). The prevalence of excessive sleepiness is higher in shift workers than in patients with obstructive sleep apnea. J. Sleep Res. 29:e13073. doi: 10.1111/jsr.13073, PMID: 32395904

[ref50] Reis D. Xanthopoulou D. Tsaousis I. (2015). Measuring job and academic burnout with the Oldenburg burnout inventory (OLBI): factorial invariance across samples and countries. Burn. Res. 2, 8–18. doi: 10.1016/j.burn.2014.11.001

[ref51] Roemer E. Schuberth F. Henseler J. (2021). HTMT2–an improved criterion for assessing discriminant validity in structural equation modeling. Ind. Manag. Data Syst. doi: 10.1108/IMDS-02-2021-0082, PMID: [Epub ahead-of-print].30908430

[ref52] Rosseel Y. (2012). Lavaan: An R package for structural equation Modeling. J. Stat. Softw. 48, 1–36. doi: 10.18637/jss.v048.i02

[ref53] Rutkowski L. Svetina D. (2014). Assessing the hypothesis of measurement invariance in the context of large-scale international surveys. Educ. Psychol. Meas. 74, 31–57. doi: 10.1177/0013164413498257

[ref54] Saboonchi F. Perski A. Grossi G. (2013). Validation of Karolinska exhaustion scale: psychometric properties of a measure of exhaustion syndrome. Scand. J. Caring Sci. 27, 1010–1017. doi: 10.1111/j.1471-6712.2012.01089.x, PMID: 23057599

[ref55] Salmela-Aro K. Rantanen J. Hyvönen K. Tilleman K. Feldt T. (2011). Bergen burnout inventory: reliability and validity among Finnish and Estonian managers. Int. Arch. Occup. Environ. Health 84, 635–645. doi: 10.1007/s00420-010-0594-3, PMID: 21082191

[ref56] Shirom A. (2003). “Job-related burnout: A review,” in Handbook of Occupational Health Psychology. eds. Quick J. C. Tetrick L. E. (Washington: American Psychological Association).

[ref57] Shirom A. Melamed S. (2006). A comparison of the construct validity of two burnout measures in two groups of professionals. Int. J. Stress. Manag. 13, 176–200. doi: 10.1037/1072-5245.13.2.176

[ref58] Sinval J. Queirós C. Pasian S. Marôco J. (2019). Transcultural adaptation of the Oldenburg burnout inventory (OLBI) for Brazil and Portugal. Front. Psychol. 10:338. doi: 10.3389/fpsyg.2019.00338, PMID: 30914985 PMC6422925

[ref59] Smets E. M. A. Garssen B. Bonke B. de Haes J. C. J. M. (1995). The multidimensional fatigue inventory (MFI) psychometric qualities of an instrument to assess fatigue. J. Psychosom. Res. 39, 315–325. doi: 10.1016/0022-3999(94)00125-O, PMID: 7636775

[ref60] Smirnova A. Y. (2017). The Oldenburg burnout inventory: diagnostics of state of Mind’s change of the employ on a continuum: work engagement – professional burnout. Philos. Psychol. Pedagogy 17, 211–218. doi: 10.18500/1819-7671-2017-17-2-211-218

[ref61] Sprinks J. (2015). Burnout among nurses. Emerg. Nurse 22:17. doi: 10.7748/en.22.9.17.s18, PMID: 25659793

[ref62] Subburaj A. Vijayadurai J. (2016). Translation, validation and psychometric properties of Tamil version of Oldenburg burnout inventory (OLBI). Procedia Soc. Behav. Sci. 219, 724–731. doi: 10.1016/j.sbspro.2016.05.067

[ref63] Tsuchiya M. Kawakami N. Ono Y. Nakane Y. Nakamura Y. Fukao A.. (2012). Impact of mental disorders on work performance in a community sample of workers in Japan: The world mental health Japan survey 2002–2005. Psychiatry Res. 198, 140–145. doi: 10.1016/j.psychres.2011.10.014, PMID: 22374551

[ref64] Vallières A. Azaiez A. Moreau V. LeBlanc M. Morin C. M. (2014). Insomnia in shift work. Sleep Med. 15, 1440–1448. doi: 10.1016/j.sleep.2014.06.021, PMID: 25277664

